# Advancing ICU mortality prediction in community-acquired pneumonia: Combining fibrinogen-to-albumin ratio, CT severity score, PSI, and CURB-65

**DOI:** 10.17305/bb.2025.12127

**Published:** 2025-02-26

**Authors:** Ece Unal Cetin, Ozge Kurtkulagi, Fatih Kamis, Murat Das, Esen Simsek, Adil Ugur Cetin, Yavuz Beyazit

**Affiliations:** 1 Department of Internal Medicine, Faculty of Medicine, Çanakkale Onsekiz Mart University, Çanakkale, Türkiye; 2 Department of Emergency Medicine, Faculty of Medicine, Çanakkale Onsekiz Mart University School of Medicine, Çanakkale, Türkiye; 3 Department of Anesthesiology and Reanimation, Faculty of Medicine, Çanakkale Onsekiz Mart University School of Medicine, Çanakkale, Türkiye; 4 Department of Internal Medicine, Çanakkale State Hospital, Çanakkale, Türkiye; 5 Department of Gastroenterology, Faculty of Medicine, Çanakkale Onsekiz Mart University, Çanakkale, Türkiye

**Keywords:** Community-acquired pneumonia, CAP, fibrinogen-to-albumin ratio, FAR, computed tomography severity score, CT-SS, pneumonia severity index, PSI

## Abstract

Community-acquired pneumonia (CAP) is a leading cause of ICU admissions, with significant morbidity and mortality. Traditional risk stratification tools, such as CURB-65, the pneumonia severity index (PSI), and computed tomography severity scores (CT-SS) are widely used for prognosis but could be improved by incorporating novel biomarkers. This retrospective study evaluated the fibrinogen-to-albumin ratio (FAR) as an additional predictor of 30-day mortality in ICU patients with CAP. A total of 158 CAP patients admitted to a tertiary care ICU were included. Baseline data encompassed demographic, clinical, laboratory, and radiological parameters, including FAR, CURB-65, PSI, and CT-SS. Logistic regression and receiver operating characteristic curve (ROC) analyses were conducted to assess mortality predictors. The 30-day mortality rate was 70.88% (112/158). Higher FAR, PSI, CURB-65, CT-SS, and lactate levels were independently associated with increased mortality (*P* < 0.05). FAR demonstrated strong discriminatory power (area under the receiver operating characteristic [AUROC]: 0.704) and significantly improved the predictive accuracy of established models. Adding FAR to PSI increased the AUROC from 0.705 to 0.791 (*P* ═ 0.009), while combining FAR, CT-SS, and PSI yielded the highest predictive accuracy (AUROC: 0.844, *P* ═ 0.032). These findings suggest that FAR, which reflects both inflammation and nutritional status, complements traditional risk assessment tools by providing a dynamic perspective. Integrating FAR into existing models enhances the identification of high-risk patients, enabling timely interventions and more efficient resource allocation in the ICU.

## Introduction

Community-acquired pneumonia (CAP) remains a major cause of morbidity and mortality worldwide, particularly among critically ill patients admitted to intensive care units (ICUs). The etiology of CAP varies by region, comorbidities, and antimicrobial resistance patterns. Common bacterial pathogens include *Streptococcus pneumoniae*, *Haemophilus influenzae*, *Mycoplasma pneumoniae*, *Legionella pneumophila*, and *Chlamydia pneumoniae*, while viral causes—such as influenza, respiratory syncytial virus (RSV), and adenovirus—are especially significant during seasonal outbreaks and in immunocompromised patients. Despite advances in antimicrobial therapy, supportive care, and preventive strategies, CAP-related mortality remains unacceptably high, highlighting the need for robust prognostic tools to guide early intervention and optimize resource allocation [1–3]. Traditional prognostic models, including the pneumonia severity index (PSI), CURB-65, and imaging-based assessments, are widely used to stratify mortality risk in CAP patients [4, 5]. However, limited data exist on the potential benefits of integrating biochemical markers—such as the fibrinogen-to-albumin ratio (FAR)—into these models, despite evidence suggesting they could enhance predictive accuracy.

The PSI and CURB-65 are well-established risk stratification tools recommended to complement clinical judgment in decision making. Both scoring systems are designed to predict short-term mortality in CAP patients [6]. PSI primarily identifies low-risk patients suitable for outpatient management, aiming to safely reduce unnecessary hospitalizations. In contrast, CURB-65 was initially developed to identify high-risk patients requiring intensive care and was later adapted to stratify patients into three severity levels, guiding management with progressively increasing intensities of medical care [4, 7]. PSI incorporates multiple clinical variables, including age, comorbidities, and laboratory findings, to estimate 30-day mortality risk. CURB-65, on the other hand, relies solely on five factors: altered mental status, urea levels, systolic blood pressure, respiratory rate, and age.

Imaging-based assessments, such as the computed tomography severity score (CT-SS), are increasingly used to evaluate lung involvement, particularly in COVID-19-related pneumonia. These assessments provide a visual measure of disease severity, which has been shown to correlate with clinical outcomes. Combining such objective metrics with biochemical markers like FAR may further improve predictive accuracy.

The pathophysiology of CAP involves a complex interplay between inflammation, infection, and the host response. Fibrinogen, an acute-phase reactant, rises during systemic inflammation, contributes to the clotting process, and plays a key role in the inflammatory cascade. Elevated fibrinogen levels have been associated with poor outcomes in various inflammatory conditions, including stroke-associated pneumonia, aortic aneurysm, and certain malignancies [8–12]. In contrast, serum albumin serves as a marker of both nutritional and inflammatory status, with its concentration decreasing in the presence of systemic inflammation and infection. Low albumin levels have been linked to adverse clinical outcomes, such as prolonged hospital stays, organ failure, and increased mortality [13]. The FAR is considered a composite marker that reflects both inflammation and nutritional status, providing a more comprehensive assessment of disease severity than either marker alone [14]. As an early serum biomarker, FAR could help identify CAP patients at high risk of in-hospital mortality, enhance prognostication, and inform ICU management and treatment decisions.

The primary objective of this study is to develop a reliable forecasting model to determine whether adding FAR to PSI, CURB-65, and CT-SS enhances their predictive performance for mortality in ICU patients with CAP. Secondary objectives include evaluating the contribution of each component to the overall model and assessing their utility in guiding clinical decision making. These findings have important implications for personalized patient care, as they could enable clinicians to tailor interventions based on a more precise assessment of mortality risk.

## Materials and methods

### Study design, definition of CAP and exclusion criteria

**Figure 1.**
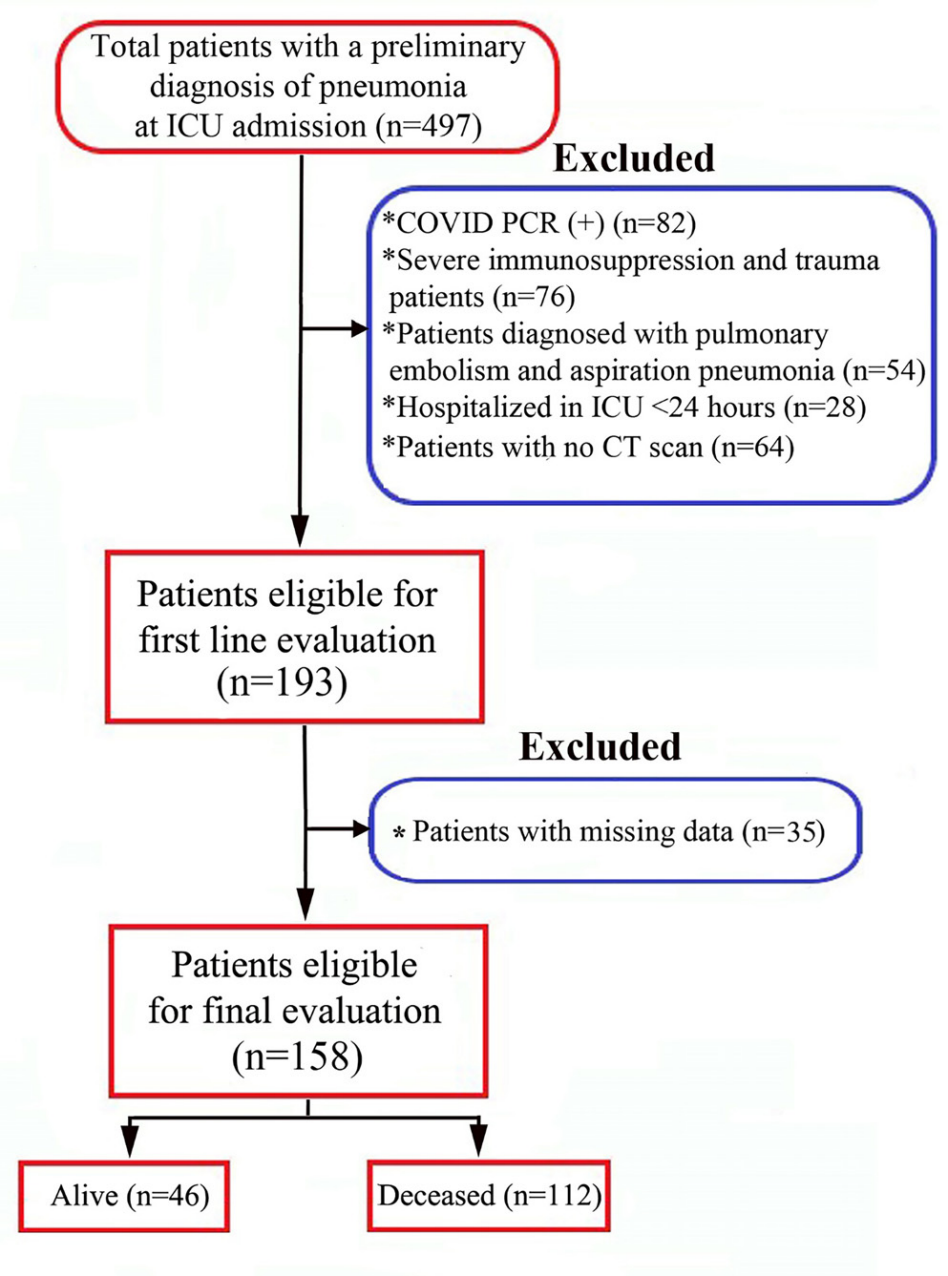
**The study flowchart of patient selection.** ICU: Intensive care unit.

All patients with CAP admitted to the Internal Medicine ICU between September 2021 and December 2023 were retrospectively analyzed (*n* ═ 497). CAP was defined as a new infiltrate on chest radiography accompanied by at least one of the following clinical signs: fever (≥38.0 ^∘^C) or hypothermia (≤36.0 ^∘^C); a new cough (with or without sputum production); pleuritic chest pain; shortness of breath; or abnormal breath sounds on auscultation. No alternative diagnosis was identified during follow-up. Patients were excluded if they were younger than 18, pregnant, diagnosed with pulmonary embolism, aspiration pneumonia, or COVID-19 pneumonia, had severe immunosuppression, experienced trauma, or were hospitalized in the ICU for less than 24 h. Additionally, patients were excluded if their diagnosis changed after treatment initiation or if a computed tomography (CT) scan could not be performed due to instability, concurrent injuries, or contraindications. Patients who did not meet the exclusion criteria were eligible for inclusion. A comprehensive flowchart outlining patient selection, recruitment, and exclusion criteria is presented in Figure 1.

### Data collection

Baseline data, including clinical, laboratory, and demographic characteristics, as well as hospital length of stay, were collected. Additionally, information on comorbidities, ICU admission sources, laboratory parameters, and CT scan findings was extracted. Cardiopulmonary parameters from the first 24 h, administered interventions (including antibiotics and mechanical ventilation), treatment protocols, and in-hospital mortality at discharge were also retrieved from the hospital’s electronic health records.

### Laboratory analysis

Hemogram and biochemical parameters—including serum glucose, total bilirubin, blood urea nitrogen, creatinine, initial serum lactate, fibrinogen, total protein, ALT, AST, and lactate dehydrogenase—were recorded for each study subject. Clinical examinations and initial laboratory tests were conducted within the first 12 h of ICU admission. All patients were monitored from admission until discharge or death.

### Screening tools to predict mortality

The clinical severity of patients was measured using four scoring systems: FAR, PSI, CURB-65, and CT-SS. FAR was calculated using SPSS statistical software by dividing the fibrinogen concentration (mg/dL) by the albumin concentration (g/L). PSI, originally proposed by Fine et al. [15], includes three demographic variables, five comorbidities, five physical examination findings, six laboratory test results, and one radiographic finding—pleural effusion. The normal PSI range is 8–90 points. Scores between 91 and 130 indicate moderate risk, while scores above 130 are associated with a high risk of mortality. CURB-65 is a six-point scoring system in which one point is assigned for each of the following criteria: confusion, urea >7 mmol/L, respiratory rate ≥30/min, blood pressure (systolic ≤90 mmHg or diastolic ≤60 mmHg), and age ≥65 years. The name “CURB-65” is derived from these criteria, with each factor contributing one point if present. CT-SS was determined based on the degree of lobe involvement in each of the five lung lobes, following the scale proposed by Chang et al. [16]. Lobe involvement was scored as follows: 0 (no involvement), 1 (<5%), 2 (5%–25%), 3 (26%–49%), 4 (50%–75%), and 5 (>75%). The total CT-SS was calculated by summing the scores of all five lobes, resulting in a final score ranging from 0 (no involvement) to 25 (maximum involvement).

### Outcome measures and mortality

The primary outcome was time to mortality within 30 days of ICU admission. For patients discharged from the hospital or who completed critical care within 30 days but lacked hospital outcome data, survival up to the 30-day mark was presumed.

### Ethical statement

This study was approved by the Çanakkale Onsekiz Mart University Ethical Committee (Approval No: 2023/14-18). Due to its retrospective design, the requirement for obtaining informed consent was waived.

### Statistical analysis

Categorical variables were summarized as frequencies and percentages (%), while continuous variables were described using the mean and standard deviation (SD). The Shapiro–Wilk test assessed the normality of continuous variables. Differences in proportions between groups were analyzed using the chi-square or Fisher’s exact test, as appropriate. The *t*-test was used to compare continuous variables between two independent groups.

Odds ratios (ORs) with 95% confidence intervals (CIs) were derived from univariate and multivariate logistic regression models to predict 30-day mortality. To examine associations between risk factor distributions and survival outcomes, multivariable Cox proportional hazards models were employed, reporting results as hazard ratios (HRs) with 95% CIs.

Receiver operating characteristic (ROC) analysis was conducted to calculate the area under the curve (AUROC) with 95% CIs for study parameters in predicting 30-day mortality. Pairwise comparisons of AUROCs were performed using the DeLong test.

All statistical analyses were conducted using SPSS version 19.0 for Windows (IBM Corp., Armonk, NY, USA) and R software version 3.6.2. A *P* value of < 0.05 was considered statistically significant.

## Results

A total of 497 CAP patients admitted to our ICU between September 2021 and December 2023 were initially enrolled. Of these, 339 were excluded for not meeting the inclusion criteria, leaving 158 patients for the final analysis (Figure 1). Among them, 85 (53.8%) were men, and 73 (46.2%) were women. The mean patient age was 75.03 ± 13.41 years (Table 1).

**Table 1:** Clinical and laboratory profiles of ICU patients with community-acquired pneumonia

**Variables**	**All patients (*n* ═ 158)**
*Demographics*	
Age (years)	75.03 ± 13.41
Gender (male, *n*%)	85 (53.8)
*ICU admission vitals*	
Heart rate (/min)	102.4 ± 25.5
Respiratory rate (/min)	21.8 ± 6.0
SBP (mmHg)	113.6 ± 25.3
MAP (mmHg)	84.3 ± 17.1
Temperature (^∘^C)	36.6 ± 0.5
*Complete blood count*	
WBC (×10^3^/uL)	14.5 ± 8.8
Hemoglobin (g/dL)	10.7 ± 2.1
Hematocrit (%)	32.8 ± 6.5
Platelet count (×10^3^/uL)	246.6 ± 140.5
*Biochemical measurements*	
Glucose (mg/dL)	171.5 ± 100.1
Urea (mg/dL)	101.9 ± 62.0
Creatinine (mg/dL)	2.04 ± 1.60
Total bilirubin (mg/dL)	0.9 ± 1.2
Fibrinogen (g/dL)	0.49 ± 0.22
Albumin (g/dL)	2.87 ± 0.6
ALT (U/L)	81.1 ± 327.4
AST (U/L)	104.6 ± 317.7
LDH (U/L)	364.6 ± 307.0
CRP (mg/L)	169.1 ± 109.4
Sedimentation rate (mm/h)	56.5 ± 31.9
Procalcitonin (ng/mL)	11.5 ± 24.4
*Illness acuity assessment tools*	
PSI	134.9 ± 33.6
CT-SS	9.7 ± 4.9
CURB-65	2.8 ± 0.9
FAR	0.181 ± 0.092
*Blood gas analysis*	
pH	7.35 ± 0.13
HCO_3_ (mmol/L)	22.2 ± 6.5
Lactate (mmol/L)	2.5 ± 2.1

PSI: Pneumonia severity index; CT-SS: Computed tomography severity score; FAR: Fibrinogen-to-albumin ratio; SBP: Systolic blood pressure; MAP: Mean arterial pressure; ICU: Intensive care unit.

The baseline characteristics of patients, grouped by survival status, are presented in Table 2. Serum urea (84.4 ± 57.4 mg/dL vs 109.2 ± 62.6 mg/dL, *P* ═ 0.010), ferritin (489.2 ± 577.5 ng/mL vs 702.6 ± 642.9 ng/mL, *P* ═ 0.007), procalcitonin (8.7 ± 24.6 ng/mL vs 12.6 ± 24.3 ng/mL, *P* < 0.001), and lactate (1.8 ± 1.5 mmol/L vs 2.8 ± 2.2 mmol/L, *P* ═ 0.002) were all significantly different between 30-day survivors and non-survivors. All severity scores were notably higher in non-survivors than in survivors: PSI (122.2 ± 35.2 vs 140.2 ± 31.6, *P* ═ 0.004), CURB-65 (2.2 ± 0.9 vs 3.0 ± 0.8, *P* < 0.001), CT-SS (7.4 ± 4.2 vs 10.7 ± 4.8, P < 0.001), and FAR (0.137 ± 0.061 vs 0.199 ± 0.098, *P* < 0.001).

**Table 2:** Prognostic factors and survival characteristics in ICU patients with community-acquired pneumonia

**Variable**	**Alive (*n* ═ 46)**	**Death (*n* ═ 112)**	***P* value**
*Demographics*			
Age (years)	72.2 ± 17.3	76.2 ± 11.3	0.150
Gender (male, %)	21 (24.7)	64 (75.3)	0.127
*ICU admission vitals*			
Heart rate (/min)	101.6 ± 24.1	102.7 ± 26.2	0.811
Respiratory rate (/min)	21.7 ± 6.4	21.8 ± 5.8	0.859
SBP (mmHg)	116.3 ± 20.9	112.5 ± 26.8	0.392
MAP (mmHg)	85.8 ± 15.2	83.7 ± 17.8	0.474
Temperature (^∘^C)	36.5 ± 0.6	36.6 ± 0.5	0.694
*Complete blood count*			
WBC (×10^3^/uL)	14.0 ± 8.3	14.7 ± 9.0	0.633
Hemoglobin (g/dL)	10.8 ± 2.4	10.6 ± 2.0	0.462
Platelet count (×10^3^/uL)	248.9 ± 122.0	245.6 ± 147.9	0.893
*Biochemical measurements*			
Urea (mg/dL)	84.4 ± 57.4	109.2 ± 62.6	0.010
Creatinine (mg/dL)	1.9 ± 1.7	2.1 ± 1.6	0.561
ALT (U/L)	70.9 ± 228.7	85.2 ± 360.9	0.804
AST (U/L)	82.4 ± 260.9	113.6 ± 338.9	0.576
Ferritin	489.2 ± 577.5	702.6 ± 642.9	0.007
CRP (mg/L)	153.4 ± 100.6	175.6 ± 112.6	0.295
Procalcitonin (ng/mL)	8.7 ± 24.6	12.6 ± 24.3	<0.001
*Illness acuity assessment tools*			
PSI	122.2 ± 35.2	140.2 ± 31.6	0.004
CT-SS	7.4 ± 4.2	10.7 ± 4.8	<0.001
CURB-65	2.2 ± 0.9	3.0 ± 0.8	<0.001
FAR	0.137 ± 0.061	0.199 ± 0.098	<0.001
*Blood gas analysis*			
pH	7.37 ± 0.11	7.33 ± 0.13	0.153
HCO_3_ (mmol/L)	22.4 ± 5.9	22.1 ± 6.8	0.353
Lactate (mmol/L)	1.8 ± 1.5	2.8 ± 2.2	0.002

PSI: Pneumonia severity index; CT-SS: Computed tomography severity score; FAR: Fibrinogen-to-albumin ratio; SBP: Systolic blood pressure; MAP: Mean arterial pressure; ICU: Intensive care unit.

In this study, we analyzed predictors of mortality using both univariable and multivariable logistic regression analyses (Table 3). Among the 158 ICU patients with CAP, the overall 30-day mortality rate was 70.9%. Higher FAR values, PSI, CURB-65, and CT severity scores (CT-SS) were all significantly associated with increased mortality (*P* < 0.05). Univariable analysis identified PSI, CURB-65, CT-SS score, FAR, urea, and lactate levels as significant predictors of higher mortality. Multivariable analysis further confirmed that FAR, CURB-65, CT-SS, and lactate remained independent predictors. Among these, FAR demonstrated the strongest association with mortality (OR 74.14 [17.74–3097.59], *P* < 0.001), underscoring its potential as a critical biomarker for assessing mortality risk in this patient population.

**Table 3:** Univariable and multivariable logistic regression analysis for the prediction of mortality in ICU patients with community-acquired pneumonia

	**Death**			
	**Univariable analysis**		**Multivariable analysis**	
	**Odds ratio (95% CI)**	***P* value**	**Odds ratio (95% CI)**	***P* value**
Age	1.022 (0.997–1.048)	0.090	–	–
Gender M (ref)	1.587 (0.796–3.166)	0.190	–	–
PSI	1.017 (1.006–1.028)	0.003	–	–
CT severity score	1.178 (1.080–1.285)	<0.001	1.197 (1.084–1.321)	0.001
CURB-65	2.531 (1.649–3.887)	<0.001	2.230 (1.331–3.736)	0.002
Procalcitonin (ng/mL)	1.007 (0.991–1.024)	0.378	–	–
FAR	20.10 (9.57–4223.04)	<0.001	74.14 (17.74–3097.59)	<0.001
Urea (mg/dL)	1.007 (1.001–1.014)	0.025	–	–
Lactate (mmol/L)	1.425 (1.087–1.868)	0.010	1.370 (1.029–1.825)	0.031
Ferritin (ng/mL)	1.001 (1.000–1.001)	0.057	–	–

PSI: Pneumonia severity index; CT-SS: Computed tomography severity score; FAR: Fibrinogen-to-albumin ratio; CI: Confidence interval; ICU: Intensive care unit.

ROC curve analysis was performed to evaluate the ability of various laboratory parameters, combined with severity scores, to predict mortality in ICU-admitted CAP patients. The optimal cut-off value for FAR to predict mortality was ≥0.160, yielding a sensitivity of 62.5% (52.9–71.5) and a specificity of 69.6% (54.3–82.3). For PSI, the best cut-off was ≥132, with a sensitivity of 60.7% (51.0–69.8) and a specificity of 63.0% (47.6–76.8), while for CURB-65, a cut-off of ≥3 resulted in a sensitivity of 79.5% (70.8–86.5) and a specificity of 63.0% (47.6–76.8). Additionally, PCT (≥ 0.9) exhibited the highest sensitivity (77.7%) with moderate specificity (56.5%), whereas CT-SS (≥8) demonstrated a strong positive predictive value (79.8%) and sensitivity (70.5%). CURB-65 (≥3) also showed robust predictive performance, with an AUROC of 0.718. These findings indicate that these parameters can serve as valuable tools for identifying high-risk patients upon ICU admission. Detailed results for all parameters are presented in Table 4.

**Table 4:** Performance of FAR, PSI, CURB-65, and CT-SS in conjunction with selected laboratory parameters for predicting 30-day mortality

	**Cut-off**	**AUROC** **(95% CI)**	**Sensitivity % (95% CI)**	**Specificity %** **(95% CI)**	**PPV %** **(95% CI)**	**NPV %** **(95% CI)**	**Accuracy % (95% CI)**
Procalcitonin (ng/mL)	≥0.9	0.711 (0.618–0.80 3)	77.7 (68.8–85.0)	56.5 (41.1–71.1)	81.3 (75.5–86.0)	51.0 (40.4–61.5)	71.5 (63.8–78.4)
FAR	≥0.160	0.704 (0.619–0.78 9)	62.5 (52.9–71.5)	69.6 (54.3–82.3)	83.3 (75.9–88.8)	43.2 (35.9–50.8)	64.6 (56.6–72.0)
Lactate (mmol/L)	≥1.6	0.660 (0.570–0.75 0)	67.0 (57.4–75.6)	52.2 (36.9–67.1)	78.0 (63.1–77.8)	39.3 (30.7–48.7)	62.7 (54.6–70.2)
PSI	≥132	0.634 (0.541–0.72 6)	60.7 (51.0–69.8)	63.0 (47.6–76.8)	80.0 (72.7–85.7)	39.7 (32.4–47.6)	61.4 (53.3–69.0)
CURB-65	≥3	0.718 (0.628–0.80 9)	79.5 (70.8–86.5)	63.0 (47.6–76.8)	84.0 (78.0–88.5)	55.6 (45.2–65.9)	74.7 (67.2–81.3)
CT-SS	≥8	0.708 (0.620–0.79 5)	70.5 (61.2–78.8)	56.5 (41.1–71.1)	79.8 (73.6–84.9)	44.1 (35.0–53.6)	66.5 (58.5–73.8)

PSI: Pneumonia severity index; CT-SS: Computed tomography severity score; FAR: Fibrinogen-to-albumin ratio; CI: Confidence interval; AUROC: Area under the receiver operating characteristic.

**Figure 2.**
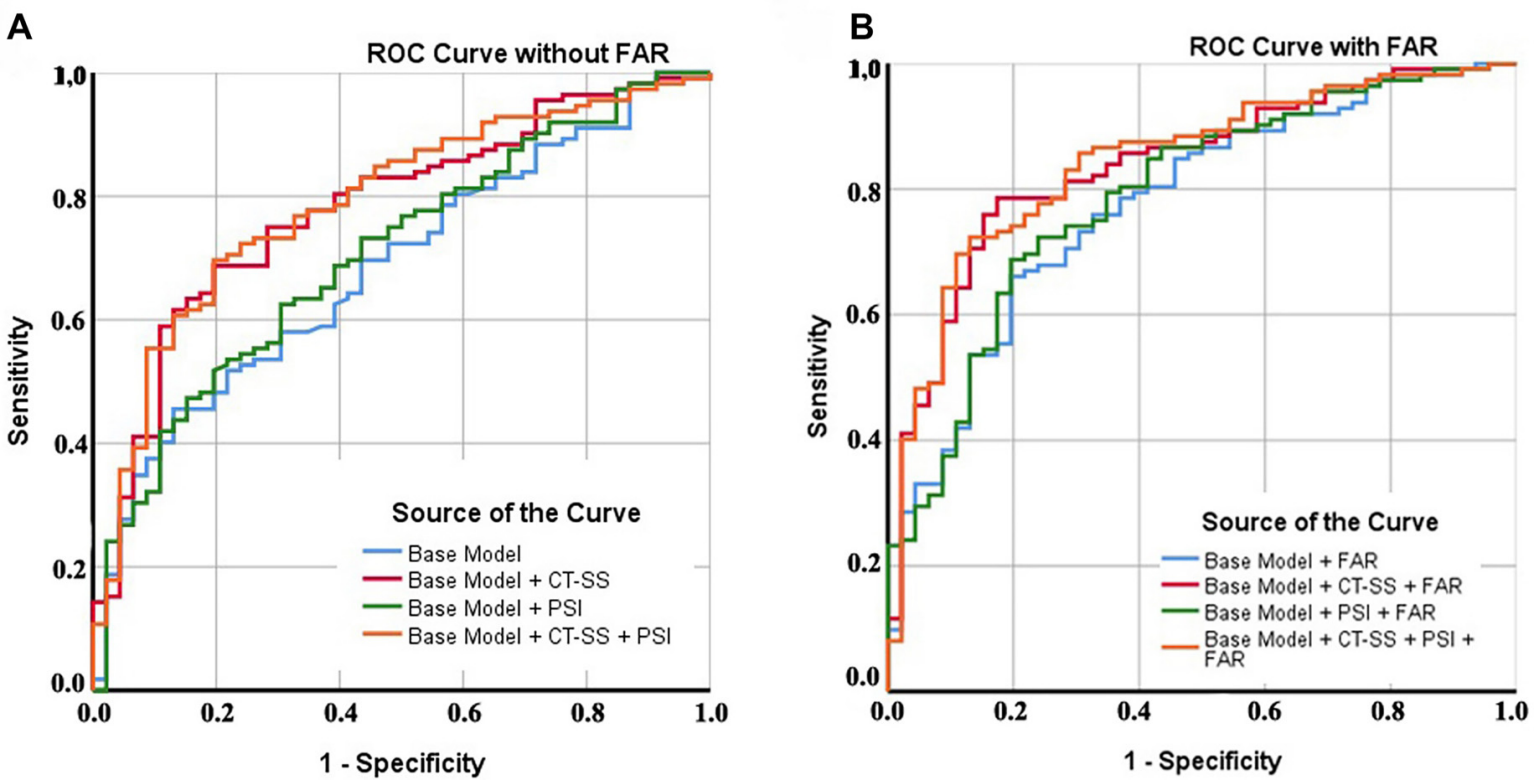
**Comparison of ROC Curves based on various predictive models with and without FAR.** (A) Without FAR; (B) With FAR. FAR: Fibrinogen-to-albumin ratio; CT-SS: Computed tomography severity scores; PSI: Pneumonia severity index; ROC: Receiver operating characteristic.

In the final step, we analyzed the impact of FAR, PSI, and CURB-65 on the discriminative accuracy of different mortality models, as presented in Table 5. Initially, we developed a base model to identify patients at high risk of mortality, considering factors, such as advanced age, male gender, and elevated lactate levels. Pairwise analysis showed that adding FAR significantly improved the discrimination accuracy of the base model (AUROC increased from 0.684–0.776, *P* ═ 0.015). Combining FAR with the base model + CT-SS and the base model + PSI also resulted in significantly higher accuracy in predicting mortality (DBA: −0.057, *P* ═ 0.037, and DBA: −0.086, *P* ═ 0.009, respectively) (Figure 2). Notably, incorporating FAR into the base model + PSI + CT-SS further enhanced predictive accuracy (DBA: −0.055, *P* ═ 0.032). These findings highlight the value of FAR in refining the predictive power of mortality models in ICU patients (Table 5).

**Table 5:** Impact of LAR, PSI, and CT-SS on the discrimination accuracy of different mortality models

	**AUROC (95% CI)**	**AUROC (95% CI)**	**Pairwise analysis**					
					**95% CI**			
**Prognostic model**	**Without FAR**	**With FAR**	**DBA**	**SE**	**Lower**	**Upper**	**Z statistic**	***P***
Base model (age, sex, lactate)	0.684 (0.597–0.771)	0.776 (0.698–0.855)	−0.092	0.287	−0.167	−0.018	−2.422	0.015
CT-SS	0.708 (0.620–0.795)	0.788 (0.713–0.863)	−0.080	0.284	−0.147	−0.014	−2.360	0.018
Base model + CT-SS	0.780 (0.703–0.857)	0.838 (0.771–0.904)	−0.057	0.267	−0.111	−0.004	−2.091	0.037
PSI	0.634 (0.541–0.726)	0.750 (0.667–0.833)	−0.117	0.296	−0.194	−0.039	−2.956	0.003
Base model + PSI	0.705 (0.619–0.791)	0.791 (0.714–0.867)	−0.086	0.284	−0.150	−0.022	−2.620	0.009
Base model + CT-SS + PSI	0.788 (0.713–0.864)	0.844 (0.778–0.909)	−0.055	0.266	−0.105	−0.005	−2.146	0.032

PSI: Pneumonia severity index; CT-SS: Computed tomography severity score; FAR: Fibrinogen-to-albumin ratio; AUROC: Area under the receiver operating characteristic; CI: Confidence interval.

**Figure 3.**
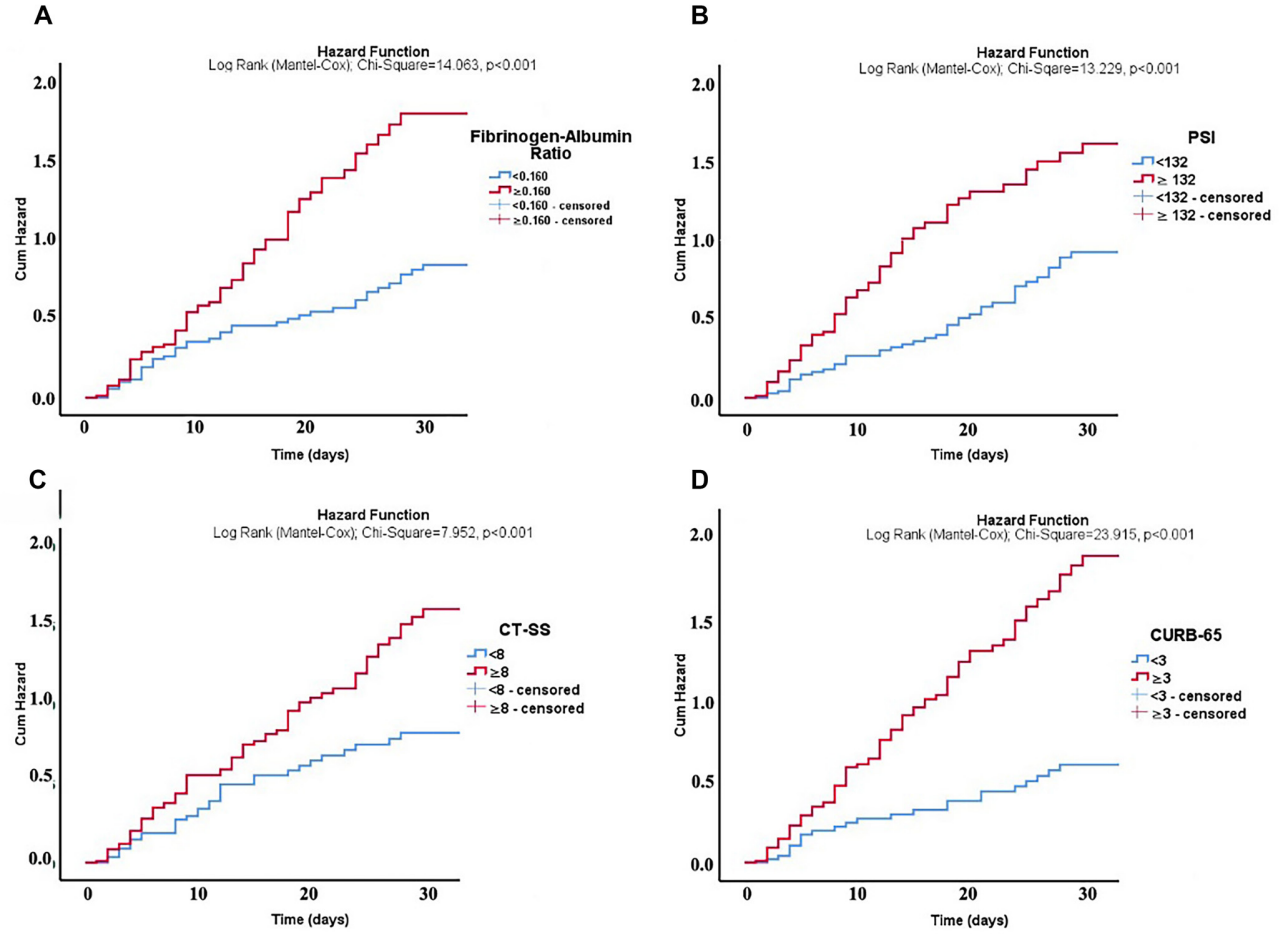
**Cumulative hazard functions for prediction of mortality based on various clinical parameters in CAP patients admitted to ICU.** (A) Fibrinogen-to-albumin ratio (<0.160 vs ≥0.160); (B) PSI (<132 vs ≥132); (C) CT-SS (<8 vs ≥8); (D) CURB-65 (<3 vs ≥3). CT-SS: Computed tomography severity score; PSI: Pneumonia severity index; CAP: Community-acquired pneumonia; ICU: Intensive care unit.

Cumulative hazard functions were analyzed to predict mortality based on various clinical parameters in CAP patients admitted to the ICU. Higher FAR (≥0.160), PSI (≥132), CT-SS (≥8), and CURB-65 (≥3) were all significantly associated with increased cumulative hazard over time (*P* < 0.001 for all comparisons) (Figure 3).

## Discussion

In this study, we explored the potential benefits of integrating FAR with PSI, CURB-65, and CT-SS to develop a more comprehensive prognostic model for ICU patients with CAP. By combining biochemical, clinical, and imaging-based measures, our goal was to address the limitations of existing models and improve mortality prediction in high-risk populations. We hypothesized that FAR, as a dynamic marker reflecting both inflammation and nutritional status, could complement the static characteristics of PSI and CURB-65, as well as the anatomical insights provided by CT-SS, to offer a more thorough assessment of patient risk.

Our findings revealed that FAR, CURB-65, and CT-SS are significantly associated with 30-day mortality, as demonstrated by both crude and adjusted multivariable logistic regression analyses. While PSI did not exhibit significant predictive capability as a standalone marker, it enhanced the prognostic performance of FAR across various models, as shown in Table 5. Additionally, FAR significantly improved the prognostic ability of CT-SS across multiple models, both with and without PSI. Incorporating FAR into PSI increased the AUROC from 0.705 to 0.791 (*P* ═ 0.009), while combining FAR with CT-SS and PSI achieved the highest performance (AUROC: 0.844, *P* ═ 0.032). These findings underscore the value of integrating a validated laboratory tool into established risk stratification systems to improve the assessment of severe critical deterioration risk in CAP patients admitted to the ICU.

The FAR is a novel biomarker that reflects the balance between systemic inflammation and nutritional status. As an emerging index, FAR has attracted significant attention in recent years due to its ability to more precisely indicate inflammatory changes. It does so by integrating the opposing trends of fibrinogen, which increases during inflammation, and albumin, which decreases. This dynamic interplay makes FAR a reliable marker for detecting and monitoring the severity of inflammatory processes. Indeed, previous studies have demonstrated its prognostic value in conditions, such as sepsis, cardiovascular disease, and malignancies [17–20]. However, its potential for predicting outcomes in ICU patients with CAP remains largely unexplored.

In this study, we demonstrated that FAR is a valuable standalone marker and that its integration into existing prognostic models significantly enhances their predictive accuracy. Our ROC analysis further confirmed FAR’s strong discriminatory power (AUROC: 0.704 [0.619–0.789]), comparable to established scores, such as PSI (AUROC: 0.634 [0.541–0.726]) and CURB-65 (AUROC: 0.718 [0.628–0.809]). Although no prior studies have examined the impact of combining FAR with other scoring systems for mortality prediction, several have highlighted its importance in CAP patients. For instance, a recent study by Luo et al. [21] found a significant increase in FAR among CAP patients, with FAR demonstrating greater predictive accuracy for CAP severity than fibrinogen alone. Additionally, FAR correlated positively with high-sensitivity CRP and the CURB-65 score. These findings suggest that FAR could be a valuable marker for assessing CAP severity and may enhance existing prognostic tools.

This study also examined the individual and combined predictive value of different scoring systems in ICU patients with CAP. Specifically, we assessed the performance of the PSI, CURB-65, and CT-SS in predicting 30-day mortality. Using ROC curve analysis, we calculated the AUC for PSI, CURB-65, and CT-SS to evaluate their ability to distinguish patients at risk of death within one month of ICU admission. For CAP patients, we found that a CURB-65 score of ≥3 and a PSI score of ≥132 were associated with a significant risk of mortality. The AUC values for predicting mortality were 0.634 (95% CI: 0.541–0.726) for PSI and 0.718 (95% CI: 0.628–0.809) for CURB-65, aligning with findings from previous studies by Gonzalez et al. [22] and Bradley et al. [23].

Additional studies have shown that CURB-65 and PSI are effective tools for predicting mortality in CAP patients [24]. Both are well-established severity scores used to assess mortality risk in CAP and help determine whether patients can be managed as outpatients. The primary strength of CURB-65 lies in its simplicity, as it relies on readily available clinical and laboratory parameters, making it an accessible tool for rapid decision making. Additionally, CURB-65 provides clear thresholds to guide clinicians in determining the need for ICU admission or more aggressive interventions, such as invasive ventilation or vasopressor support. However, despite its utility, CURB-65 primarily focuses on physiological and demographic factors, without accounting for comorbidities or radiological findings that could influence CAP outcomes [24–26]. Conversely, PSI is a more comprehensive scoring system that incorporates demographic information, comorbidities, vital signs, laboratory values, and radiological findings to generate a point-based score. It classifies patients into five risk categories, with higher scores indicating greater mortality risk. Unlike CURB-65, which primarily identifies patients at high risk of mortality, PSI is designed to pinpoint those at low risk and offers a more nuanced assessment, particularly for chronic conditions like liver or renal disease [24]. However, PSI is more complex, requiring additional time for data collection and calculation, making it less practical in resource-limited settings. Studies have shown that higher PSI scores correlate with an increased risk of complications, such as septic shock and multi-organ failure, underscoring its predictive value [27, 28].

The CT-SS complements CURB-65 and PSI by evaluating pulmonary involvement through radiological imaging. It scores lobe involvement from 0 (no involvement) to 25 (maximum involvement), providing a direct quantification of lung involvement—something CURB-65 and PSI do not offer. Higher CT-SS scores correlate with severe hypoxemia, heightened inflammatory burden, and increased mortality risk. However, data on CT-SS in CAP remains limited, with most studies focusing on COVID-19 pneumonia [29, 30]. This gap underscores the significance of our study, as incorporating CT-SS offers a visual and measurable parameter for disease progression. Notably, we identified a CT-SS of ≥8 as a threshold for increased mortality risk, with a specificity of 56.5% and a sensitivity of 70.5%. Similarly, Bardakci et al. [31] proposed a cut-off level of >10 for COVID-19 pneumonia patients, reporting a specificity of 79.7% and a sensitivity of 82.3% (AUROC: 0.708 [0.620–0.795], sensitivity: 70.5% [61.2–78.8], specificity: 56.5% [41.1–71.1]).

Although this study underscores the importance of using risk stratification tools in combination, its findings should be interpreted with caution due to certain limitations. First, its retrospective design may introduce selection bias, and external validation in larger, more diverse cohorts is needed to ensure generalizability. Second, the dynamic nature of CAP progression necessitates further exploration of temporal changes in FAR and other parameters to enhance predictive accuracy. Finally, as the study was conducted at a single tertiary care center, the relatively small sample may not fully represent the general population.

## Conclusion

In conclusion, integrating the FAR with established clinical and radiological scores, such as PSI, CURB-65, and CT-SS enhances the accuracy of mortality prediction in ICU patients with CAP. The inclusion of laboratory markers like FAR in existing models provides a more comprehensive approach to risk stratification, facilitating timely and informed decisions on resource allocation in critical care settings.
